# Case Report: Good Prognosis of Mixed Alien Hand Syndrome by Verbal-Cue Rehabilitation Exercise

**DOI:** 10.3389/fneur.2021.718706

**Published:** 2021-09-08

**Authors:** Kang Qu, Lin Gan, Wei Jiang, Peng Yu, Ming Dong

**Affiliations:** ^1^Department of Neurology and Neuroscience Center, The First Hospital of Jilin University, Changchun, China; ^2^Department of Ophthalmology, The Second Hospital of Jilin University, Changchun, China

**Keywords:** alien hand syndrome, prognosis, cerebral infarction, corpus callosum, case report

## Abstract

Mixed alien hand syndrome is a rare disease reported in the literature. The mixed callosal–frontal variant of alien hand syndrome is associated with uncoordinated hand movements, and patients may present with an involuntary grasp reflex and intermanual conflict. There are few videos in the existing literature on the comparison of patients' condition before and after recovery of the symptoms of mixed alien hand syndrome. We presented the prognosis of mixed alien hand syndrome in the form of a video. In addition, we have included some videos on the comparison of the condition of patients before and after recovery of the symptoms of mixed alien hand syndrome. A 57-year-old woman presented with left-handed intermanual conflict and right-handed involuntary grasp reflex due to infarction of the frontal lobe and corpus callosum. She was diagnosed with a mixed callosal–frontal variant of alien hand syndrome. Her left hand counteracted the purposeful movements of the right hand. However, the intermanual conflict disappeared after 3 months of therapy, including drug treatment and verbal-cue rehabilitation, and she regained normal coordination of her hand movements. Her prognosis was good despite the large corpus callosum lesions. The uncoordinated hand movements of the patient affected her daily life and caused psychological problems. Initiating rehabilitation early was important and necessary for her to regain coordination. It is possible that the verbal-cue training method played an important role in the recovery of the patient. Therefore, this method of rehabilitation deserves consideration and can be adopted in larger cohort studies as we presented only a single case. The possible mechanisms behind the verbal-cue exercise require further studies, and this patient had a good prognosis despite severe corpus callosum injury, which may merit further investigation.

## Introduction

Alien hand syndrome (AHS) mainly manifests as uncontrollable involuntary movements of the affected hand, and patients often describe the feeling that their hand is not under their control. In addition, patients also present with intermanual conflict and denial of the disordered hand as being part of their body (1). This disorder usually involves the left hand and is described as “hand-anthropomorphic.” At present, AHS is believed to occur due to cortical basal ganglia degeneration, stroke, tumor, and degenerative brain disease (2). However, cases of mixed variants are very rare, and the prognosis of mixed AHS is unclear. Here, we report a case of a mixed callosal–frontal variant of AHS with a good prognosis, even when associated with extensive corpus callosum damage.

We present the following case in accordance with the CARE reporting checklist.

## Case Presentation

A 57-year-old right-handed woman presented with involuntary compulsive grasping with her right hand and intermanual conflict with her left hand. The typical symptom of involuntary compulsive grasping and grabbing with her right hand, which was associated with a frontal variant of AHS, manifested in this patient as an inability to release a comb or the hand of the examiner during the muscle strength examination (Supplementary Video 1). In addition, the typical intermanual conflict associated with the callosal variant of AHS occurred while she combed her hair or ate food with her right hand; her left hand counteracted the purposeful movements of her right hand (Supplementary Video 1). However, her hand movements were perfectly coordinated and smooth when she used her left hand for purposeful movement. She said that these symptoms, which were not under her control, affected her daily life. She presented to our hospital because she could not control her left hand. She had not received any medical treatment for this condition until she was admitted to our hospital. She had a history of hypertension. A history of epileptic seizures, cranial demyelinating pathologies, and other neurodegenerative diseases was not elicited. In addition to antihypertensive drugs (calcium channel blocker), there was no other medication history and no relevant family history. The general examination showed no obvious abnormalities, and her vital signs were normal. Neurologic examination revealed positive Babinski and Chaddock signs in her right lower extremity, and the muscle power of the right limbs was 4/5 grade. The grasp reflex was positive. The rest of the physical examination disclosed no abnormalities. Her National Institutes of Health Stroke Scale score was 3 points. Routine laboratory test results were normal.

**Figure 1:**
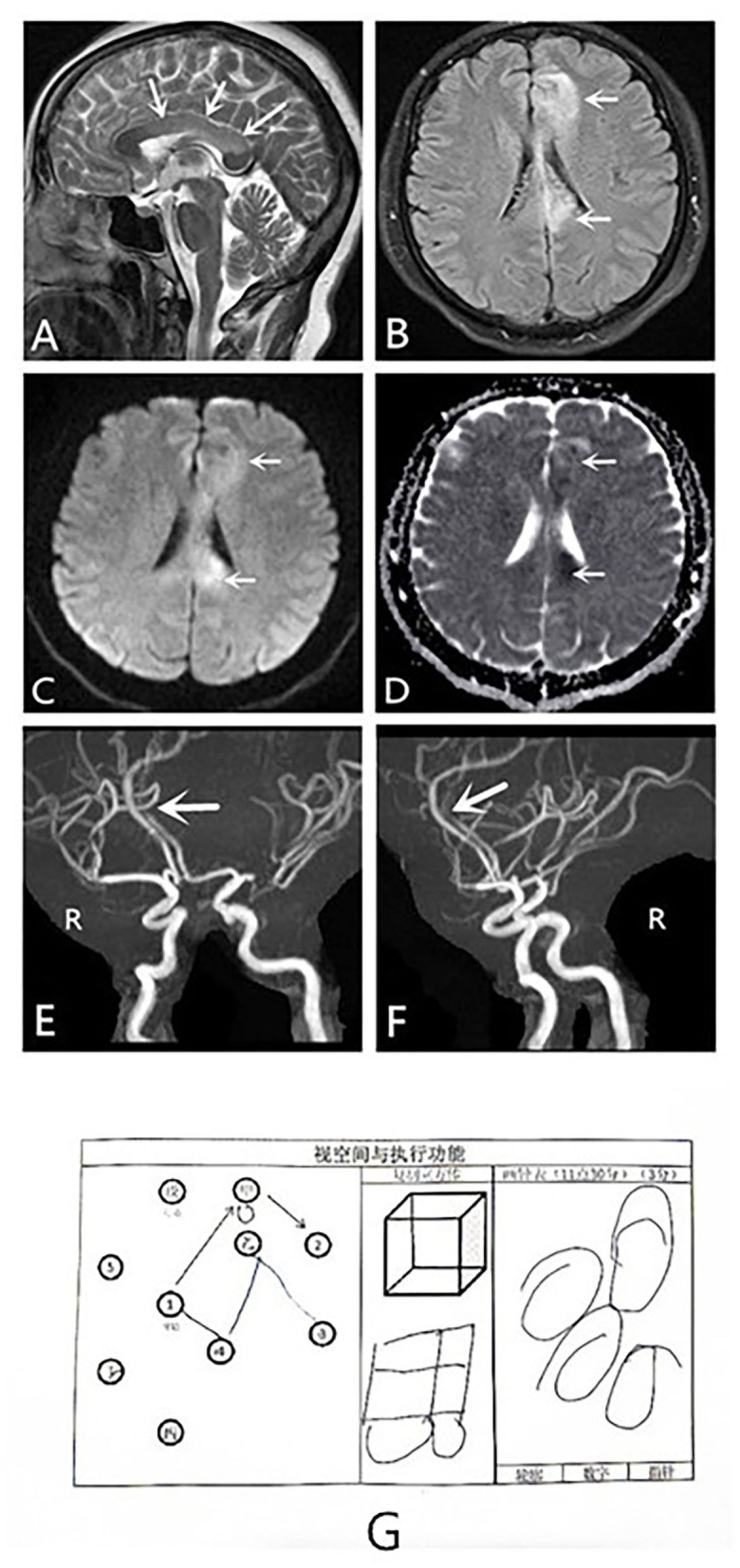
MRI, MRA and visuospatial and executing skills of the dominant hand results of a patient with alien hand syndrome. MRI: **(A–D)** Sagittal T2WI scan, axial T2 FLAIR, DWI, and ADC scans show cytotoxic edema in the left frontal lobe and the body of the corpus callosum. (**E,F**) MRA shows poor visualization of the pericallosal artery and stenosis of the left middle cerebral artery. The patient failed to recognize spatial structure, known as constructional apraxia **(G)**. ADC, apparent diffusion coefficient; DWI, diffusion-weighted imaging; FLAIR, fluid-attenuated inversion recovery; MRA, magnetic resonance angiogram; MRI, magnetic resonance imaging; T2WI, T2-weighted imaging.

Magnetic resonance imaging (MRI) revealed that she had developed ischemic strokes in the corpus callosum and the left frontal lobe due to stenosis of the A4 segment of the anterior cerebral artery. MRI revealed cytotoxic edema in the left frontal lobe and the body of the corpus callosum. The infarct involved approximately two-thirds of the corpus callosum, as observed on the sagittal T2-weighted image (T2WI) (Figures 1A–D). Magnetic resonance angiography (MRA) revealed poor visualization of the A4 segment of the left anterior cerebral artery (pericallosal artery) and stenosis of the left middle cerebral artery (Figures 1E,F).

Therefore, she was diagnosed with a mixed callosal–frontal variant of AHS. We considered the cause of her AHS to be ischemic strokes in the corpus callosum and left frontal lobe. The patient was administered routine aspirin antiplatelet therapy (100 mg QD) and verbal-cue rehabilitation to assist her in correcting the conflicting movements of her hands early for movement coordination. Whenever the patient performed an intentional movement with her right hand, such as combing her hair or eating with her right hand, her left hand interfered and prevented the movements of the right hand. This condition affected almost all movements that required coordination between her hands. Whenever she showed intermanual conflict in her left hand, her guardian verbally prompted her with instructions to complete the movement she wanted to perform correctly. Verbal cues were patiently used until the patient corrected her discordant movements herself. We found verbal cues a method to correct intermanual conflict in her left hand to be timely and effective, as can be observed in Supplementary Video 1. With each repetition of verbal cues during rehabilitation training, the frequency of her intermanual conflict symptoms gradually decreased. The patient reported a confidence in herself and the training method and hoped for a speedy recovery. Her guardian said there was no rebound of the intermanual conflict symptoms as the frequency of verbal cues decreased. After 3 months, the intermanual conflict in her left hand disappeared as did the grasp reflex of her right hand. The movements between her hands became coordinated and orderly. Her guardian reported that her left hand no longer interferes with her right hand while she combs her hair, eats, or puts on clothes with her right hand. Her hand movements became as smooth and coordinated as those before symptom onset (Supplementary Video 2). At the follow-up visit, after the strength test, she successfully released the hand of the examiner voluntarily instead of holding it tightly. Irrespective of whether she combs her hair with her left or right hand, the movements are coordinated and orderly, as can be seen in Supplementary Video 2.

**Figure 2:**
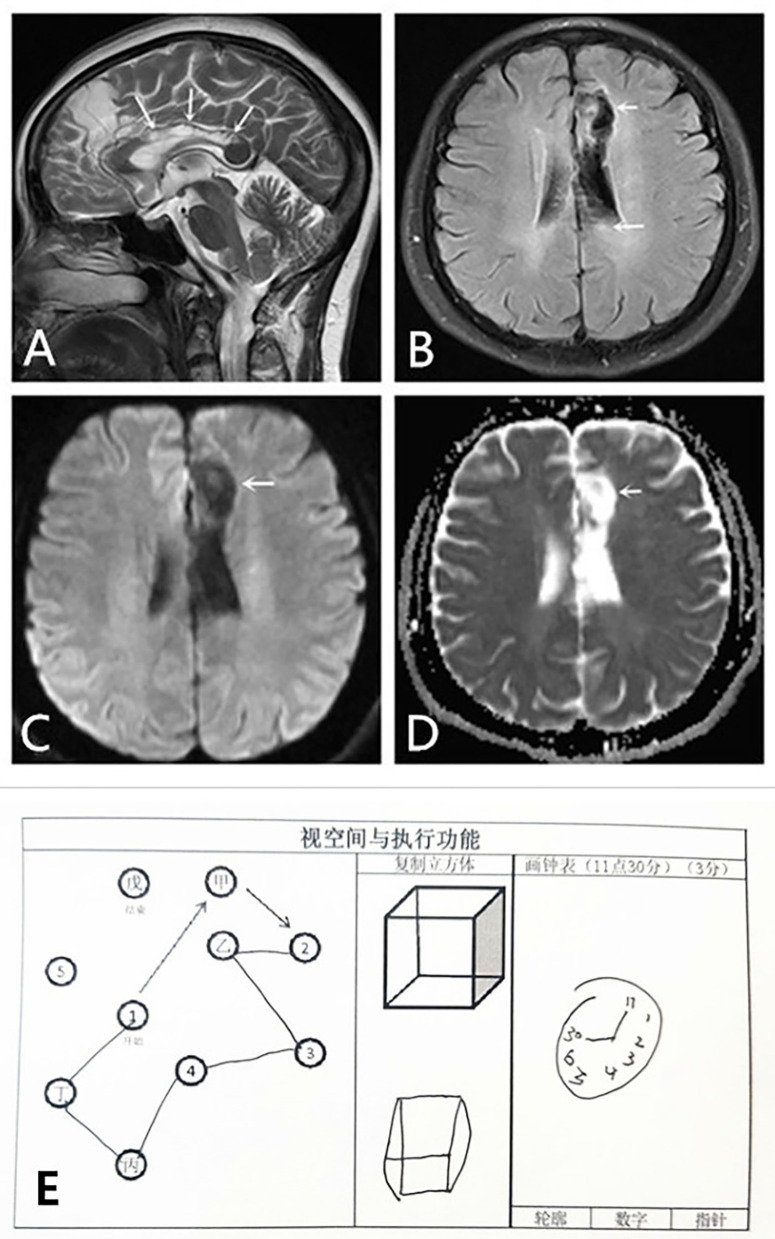
Cytotoxic edema and visuospatial and executing skills of the dominant hand of the patient improved in 3 months. MRI: **(A,B)** Sagittal T2WI scan and axial T2 FLAIR show obvious improvement of cytotoxic edema in the left frontal lobe and the body of the corpus callosum. Encephalomalacia foci and gliocyte hyperplasia can be observed in these areas. **(C,D)** DWI and ADC scans show no limitation of diffusion. The patient failed to recognize spatial structure; however, she has made some progress after this period of recovery **(E)**. ADC, apparent diffusion coefficient; DWI, diffusion-weighted imaging; FLAIR, fluid-attenuated inversion recovery; MRI, magnetic resonance imaging; T2WI, T2-weighted imaging.

She also had a symptom of constructional apraxia of the dominant hand. She could feel shapes, distinguish objects, and locate them, but she could not identify the stereostructure, which is characteristic of the callosal variant of AHS. For example, she could not correctly complete drawing a clock and imitate a square schematic to draw a square (Figure 1G). On the return visit of the patient, she still could not correctly identify the stereostructure (Figure 2E). However, she has made some progress after a period of recovery.

The compliance of the patient to the intervention was good, and there was effective and timely correction of her motor coordination. The patient also showed apathy because of the discordant movements of the hands. Therefore, we assessed her compliance with this intervention based on her acceptance and the Modified Apathy Evaluation Scale (MAES) scoring scale. Her frustration lessened as the training progressed, and the MAES score gradually decreased from 20 to 8. This method of rehabilitation requires no special training, as long as the patient and guardians patiently cooperate with each other. There were no adverse or unexpected events during the intervention.

At the same time point, the sagittal T2WI and axial T1 fluid-attenuated inversion recovery (FLAIR) image showed obvious improvement in the cytotoxic edema of both the left frontal lobe and body of the corpus callosum (Figures 2A,B). Diffusion-weighted imaging and apparent diffusion coefficient scans showed no limitations of diffusion (Figures 2C,D). Despite improvement of the lesion in the left splenium of the corpus callosum, the lesions in the left frontal lobe and the body of the corpus callosum were persistent and more remarkable than before (Figure 2A).

All procedures performed in this study involving human participants were in accordance with the ethical standards of the institutional research committee and with the Helsinki Declaration (as revised in 2013). Moreover, this study involving human participants was reviewed and approved by the ethics committee of the First Hospital of Jilin University. Written informed consent was obtained from the patient.

## Discussion

AHS is a rare group of clinical manifestations mainly characterized by limb movement disorders, which involves three variants: frontal, callosal, and posterior (1). The frontal variant of AHS, characterized by involuntary groping and difficulty in releasing after grasping, is usually caused by a lesion in the unilateral frontal lobe with or without the involvement of the corpus callosum and mostly affects the dominant hand (2). The patient in this report presented with a typical grasp reflex in her right hand, which was caused by a left frontal lobe and corpus callosum stroke due to ischemia of the anterior cerebral artery. Lesions in the anterior corpus callosum, the supplementary motor area of the frontal lobe, the cingulate gyrus, or the anterior prefrontal cortex can lead to the loss of inhibitory control of the motor cortex on the affected side, the inability to transfer inhibitory signals to the contralateral side to bring about the releasing movement from the inhibitory control of the contralateral cortex, and interaction disorder between the frontal and parietal lobes (3). A frontal lobe lesion affects avoidance behavior, and the release of information from the parietal lobe leads to the relative excessive release of approach behavior, thus, causing the gripping symptoms of the affected hand (4). The callosal variant of AHS is characterized by intermanual conflict and is usually caused by a lesion in the corpus callosum (5). It can also be accompanied by symptoms of apraxia, such as competitive or constructional apraxia. The corpus callosum, which relays information between the bilateral cerebral hemispheres, contains the majority of the interconnecting fibers of the frontal cortex to coordinate bilateral synchronized movements and allow orderly dissociation during bilateral actions that require coordinated but different movements on each side (6). When the corpus callosum is damaged, the connection between the bilateral cerebral hemispheres is severed, and inhibitory information sent by the contralateral cortex is lost (3). The failure to integrate information between the two cerebral hemispheres might lead to uncoordinated hand movements, and symptoms such as interhemispheric separation might be present (7).

She also had a symptom of constructional apraxia of the dominant hand. Apraxia can be attributed to lesions in the supplementary motor area or the prefrontal and parietal cortices of the dominant hemisphere (8). Lesions of the corpus callosum can cause disconnection between hemispheres and affect the transfer of exercise information from the dominant to the non-dominant hemisphere (9).

Accordingly, the intermanual conflict in the left hand of this patient was caused by a lesion in the corpus callosum, while the involuntary compulsive grasping in her right hand was caused by lesions in the left frontal lobe and the corpus callosum. Thus, she was diagnosed with mixed AHS.

These symptoms, which were not under her control, led to uncoordinated movements of her hands, which affected her daily life and caused psychological problems (Modified Apathy Evaluation Scale, 20 points).

The corpus callosum receives blood supply from three main arterial systems: the anterior cerebral, anterior communicating, and posterior cerebral arteries. Corpus callosum infarction is rarely seen clinically because of its abundant blood supply (10). In this case, MRA revealed poor visualization of the pericallosal artery, which is the A4 segment of the left anterior cerebral artery. The pathophysiology of AHS can be associated with supplementary motor area lesion formation, interhemispheric disconnection, or disorders of volitional movement and motor control (3). Thus, an ischemic lesion in the corpus callosum may lead to failure to integrate the movement information of the bilateral coordination of synchronous movements.

At present, therapies for AHS generally include mirror box therapy, visuospatial coaching techniques, distracting the affected hand, goal-oriented training, and other physical and cognitive therapies (11). Botulinum toxin injection and clonazepam treatment have also achieved good results in some studies (12). There are no approved treatments in the field of rehabilitation of the alien hand. In the literature, most of the available treatments are individualized approaches, and there are relatively few data on the rehabilitation of patients with alien hand. Therefore, we report an individualized treatment protocol to provide a reference for clinicians and to facilitate the discussion of the best treatment options for alien hands.

The frontal lobe and large corpus callosum lesions resulted in mixed AHS. The supplementary motor area (SMA), located in the medial frontal lobe, is connected with the primary motor cortex and contralateral SMA and is involved in motor planning, initiation, and inhibition (13). Its lesions lead to an absence of planning and disorder of movement. Lesions in the SMA can also cause the loss of the original automatic inhibition response to the environment, which explains the grasping reflex seen in AHS (14). The pre-SMA participates in the conscious experience function of voluntary movement, allowing the body to understand the action intention of the voluntary movement (8). Consciousness is closely linked to the control of movement (1). The corpus callosum has abundant connective fibers with the frontal cortex. The corpus callosum is responsible for the integration and communication of motor information between the two hemispheres, so that the movements that need bilateral cooperation can be coordinated and separated in an orderly and smooth manner (15). When there is a loss of connection between the hemispheres, inhibitory information is not conveyed, resulting in the loss of motor coordination (3). We found that the guardian of the patient could effectively coordinate the movements of the hands of the patient through verbal cues, with the purpose of controlling the alien hand. This can be proved through Supplementary Video 1, which suggests the possibility of using verbal aids to inhibit the uncoordinated behavior of the alien hand. This is consistent with the findings of Cantagallo et al. (11). We suggest that external verbal cues may help patients reprogram their movements and, thus, improve their ability to control the alien hand. Verbal cues can help patients develop a stronger sense of control over volitional movements and can also guide the alien hand toward a visual target in the correct sequence. When the visual feedback to the visual target was consistent with the motor intention, the control of voluntary movement was improved. Romano et al. validated this theory that motor intent consistent with visual feedback information can control the alien hand using a mirror box model (16). Repetitive verbal cues activated the contralateral SMA. We speculate that through this repeated training with verbal instructions, patients may develop self-correcting reflexes.

Our patient had a good recovery of symptoms following 3 months of antiplatelet therapy and verbal-cue rehabilitation training. The intermanual conflict disappeared after 3 months of rehabilitation training, and the hand movements were coordinated and orderly. The sagittal T2WI and axial T1 FLAIR image showed obvious improvement in cytotoxic edema in both the left frontal lobe and body of the corpus callosum over the course of 3 months. However, despite the improvement in the lesion in the left splenium of the corpus callosum, the lesions in the left frontal lobe and the body of the corpus callosum persisted. The improvement of the symptoms of the patient might have been partially spontaneous due to the substantial improvement in cytotoxic edema in the body of the corpus callosum. The pericallosal arterial plexus is composed of the anastomotic branches of the supplying arteries in the corpus callosal sulcus, and abundant collateral circulation can be established rapidly after vascular occlusion (9). If the infarct is one-sided or partial, the remaining healthy tissue may compensate for the impaired tissue function and reestablish the connection between the two hemispheres in a short period. The parts of the corpus callosum without lesions may have compensated greatly for the affected regions, and the integration of movement information between the bilateral cerebral hemispheres was successfully reestablished (17).

It is possible that the verbal-cue training method played an important role in the recovery of the patient. Therefore, this method of rehabilitation deserves consideration and needs to be adopted in larger cohort studies as we presented only a single case. The possible mechanisms behind the verbal-cue exercise require further studies using functional magnetic resonance imaging combined with diffusion tensor imaging and other techniques.

## Conclusions

A special feature of our case is that the prognosis of the patient for mixed callosal–frontal variant AHS was good following 3 months of antiplatelet therapy and verbal-cue rehabilitation training despite her large corpus callosum lesions. We shared both the rare and typical clinical symptoms of mixed AHS in videos, which may provide clinicians with a greater ability to make quick and accurate diagnoses and serve as an important reference for clinical prognosis. Further studies into better treatment of AHS should utilize structural and functional imaging. Moreover, the reasons for the good prognosis of AHS deserve further investigation in larger cohort studies.

This case report aims to raise awareness of this rare disease and provide a reference for its treatment and prognosis.

## Data Availability Statement

The original contributions presented in the study are included in the article/Supplementary Material, further inquiries can be directed to the corresponding author/s.

## Ethics Statement

Written informed consent was obtained from the individual(s) for the publication of any potentially identifiable images or data included in this article.

## Author Contributions

KQ contributed to the conception and design of the study and drafting of the manuscript. LG and WJ contributed to the acquisition of the data. PY and MD contributed to the conception, design, and critical revision of the manuscript for intellectual content. All authors read and approved the final version of the manuscript.

## Funding

This work was supported by the National Natural Science Foundation of China (Grant No. 31872772 to MD) and the Natural Science Foundation of Jilin Province of China (Grant No. 20200201606JC to MD).

## Conflict of Interest

The authors declare that the research was conducted in the absence of any commercial or financial relationships that could be construed as a potential conflict of interest.

## Publisher's Note

All claims expressed in this article are solely those of the authors and do not necessarily represent those of their affiliated organizations, or those of the publisher, the editors and the reviewers. Any product that may be evaluated in this article, or claim that may be made by its manufacturer, is not guaranteed or endorsed by the publisher.
